# The Landscape of the Gibbet

**DOI:** 10.1080/01433768.2015.1044284

**Published:** 2015-04-30

**Authors:** Sarah Tarlow, Zoe Dyndor

**Keywords:** Gibbet, criminal, execution, body, eighteenth century, Britain

## Abstract

From the Murder Act of 1752 until the Anatomy Act of 1832 it was forbidden to bury the bodies of executed murderers unless they had first been anatomised or ‘hung in chains’ (gibbeted). This paper considers some of the observations of the Wellcome-funded project ‘Harnessing the Power of the Criminal Corpse’ as they relate to the practice of gibbeting. The nature of hanging in chains is briefly described before an extensive discussion of the criteria by which gibbets, which often remained standing for many decades, were selected. These are: proximity to the scene of crime, visibility, and practicality. Exceptions, in the forms of those sentenced by the Admiralty Courts, and those sentenced in and around London, are briefly considered. Hanging in chains was an infrequent punishment (anatomical dissection was far more frequently practised) but it was the subject of huge public interest and attracted thousands of people. There was no specified time for which a body should remain hanging, and the gibbet often became a known landmark and a significant place in the landscape. There is a remarkable contrast between anatomical dissection, which obliterates and anonymises the body of the individual malefactor, and hanging in chains, which leaves a highly personalised and enduring imprint on the actual and imaginative landscape.

## INTRODUCTION

This paper discusses some of the findings of the interdisciplinary research programme ‘Harnessing the Power of the Criminal Corpse’ funded by the Wellcome Trust. It focuses on the public exhibition of the bodies of executed criminals between 1752 and 1832, and outlines the factors that informed the siting of gibbets as well as considering how the presence of a gibbet could affect the landscape at the time of its erection and for the long years afterwards. Finally it considers the legacy of the gibbet in the contemporary landscape.

This paper largely supports a Foucauldian interpretation insofar as it sees the materiality of both the body and the landscape as a distinctive feature of punishment in the long eighteenth century, in distinction to a more ‘modern’ reformative punishment concerned with reclaiming the intangible and abstract ‘soul’ of the criminal. As a post-mortem punishment that depended on the display of the dead body as a vain and abject thing, deprived of its animating divine spark, hanging in chains also draws upon a deep tradition of Christian discourse that contrasts the eternal soul with the ephemeral and vain body. It is undue attention to the needs and desires of the latter that causes sin; and there is therefore a pleasing symmetry to practices that punish sin in the body responsible for its commission. Where fleshly sins such as, most commonly, lust, greed, envy or anger led a person to break the fifth commandment, the flesh suffers the punishment; through ostentatious display of that dismal flesh, the vanity of bodily gratification, rather than attention to one's immortal soul, is publicly pronounced.

In fact, we argue here, the line between shaming notoriety and immortal celebrity is thin, and an unintended consequence of what was often considered a display of social power, a religious lesson and a means of restoring the balance and moral health of the community is that the criminal acquired a permanent legacy in the landscape. This was both material — in the form of a prominently positioned gibbet that endured for decades — and mnemonic, in placenames, songs, stories and local memory.

There is much more that could be said about the history, folklore and social impact of the gibbet, much of which will be developed in future publications. This article concentrates specifically on its landscape dimensions. An attempt will first be made to determine what factors were significant in the selection of gibbet locations across England and Wales. It has been suggested that gibbets were placed on parish boundaries (Whyte 2003) and on highways (Gatrell 2004). While there were gibbets placed on such sites, these were not the only places on which gibbets were situated, and a variety of considerations were involved in selecting the gibbet location. Finally the paper will consider the legacy of the gibbet in the contemporary landscape of which, with the exception of Whyte (2003), there has been no scholarly analysis. We make extensive use of the data collected for the Wellcome Trust funded research programme ‘Harnessing the Power of the Criminal Corpse’. The project has focused on the ways that the body of the executed criminal was powerful, especially during the core period between the Murder Act and the Anatomy Act. As there is no single source detailing what the post-mortem punishment of murders was, primary data was collected from a variety of sources to determine the fate of the body of each murderer in England and Wales between 1752 and 1834. The most significant source used was the little known ‘Sheriffs’ Cravings’ in the National Archives at Kew. These are the expense claims made by sheriffs whose job it was to organise the keeping of prisoners, facilitate assize sittings and carry out sentences. These itemised claims record, often in considerable detail, the expenditures necessary to ensure that an executed criminal body was buried, dissected or hung in chains (Ward forthcoming). In addition, a sample of assize records was taken for each circuit, and then systematically checked for those circuits in which details of the post-mortem punishment were recorded. Both local and national newspapers were searched for references to gibbets and the name of every murderer, as were periodicals and online databases such as ECCO. County record offices were visited to search for references relating to the crime, criminal and details of post-mortem punishments. Using this data, a project database has been compiled containing information on each murderer and the post-mortem punishment they received. All figures in the article have been derived from the project data unless otherwise stated.^1^

**Fig. 1.**
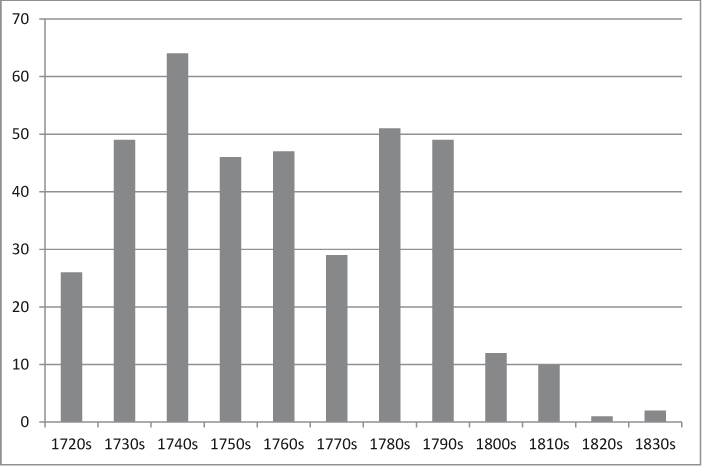
The frequency of hanging in chains by decade

Gibbeting (or ‘hanging in chains’ as it is called in most literature of the period) was never the most widely practised post-mortem punishment and even at its peak in the mid-eighteenth century was a comparatively rare occurrence; many counties had fewer than five gibbetings in the whole eighty-year period and some had none at all. The frequency of hanging in chains by decade (in England and Wales) is represented by Fig. 1. Hanging in chains in fact reached a peak before the Murder Act, during the 1740s, and the practice declined sharply at the end of the eighteenth century. In 1832 there were two final incidences, both during the summer after the passage of the Anatomy Act, and probably responses to a misreading of the terms of that Act, which specified that the bodies of executed murderers should no longer be given to the anatomists, whose needs were now to be supplied by the bodies of those dying ‘unclaimed’ in workhouses and hospitals. The alternative provision for murderers — that they could be buried within the prison precinct — was perhaps missed, leaving the impression that the only recourse was the gibbet. In fact both of these cases — William Jobling in Jarrow and James Cook in Leicester — were the focus of massive public interest and press disapproval. Authorities were concerned by the large numbers of people who came to view the bodies and subsequent disruption caused. Neither of these bodies remained on the gibbet for very long and the sentence was not passed again. The practice was formally abolished in 1834.

The period is bookended by two significant parliamentary acts. In 1752 the Act for Better Preventing the Horrid Crime of Murder sought to distinguish murder as a crime of particular horror by added post-mortem provision as a compulsory part of sentencing. All hanged murderers were to be denied church burial, unless they had been first either dissected by anatomists or hung in chains. The Act was only superseded in 1832 by the Anatomy Act, a response to the public fear of resurrectionists and body trade scandals such as the much publicised Burke and Hare affair (Douglas 1973; Bailey 2002). The Anatomy Act formally ended the dissection of criminal bodies, substituting the more plentifully available bodies of the ‘unclaimed’ poor (Richardson 1989; Hurren 2012). Hanging in chains was formally abolished two years later, although very few gibbetings had taken place after the first few years of the nineteenth century. In fact our research shows that only twenty-five men were gibbeted between 1800 and the abolition of the Murder Act.

The majority of those sentenced for killing offences in England and Wales in the period 1752 to 1832 were sentenced to dissection. Analysis undertaken as part of the Criminal Corpse project shows that in total 79 *per cent* (908) of the 1,151 offenders capitally convicted for killing offences in this period were punished in this way.^2^ Of the remaining 21 *per cent*, 8 *per cent* were pardoned from execution, less than 1 *per cent* died in prison before their execution and the remaining nearly 13 *per cent* were ‘hung in chains’ — 148 individuals over this eighty-year period. Alongside these men gibbeted under the Murder Act (and all gibbetted individuals in this period were men; women convicted under the Act were invariably sent for anatomical dissection), a further seventy-five individuals were subjected to this post-mortem punishment for various forms of robbery, arson and riot. These were largely for mail robbery and highway robbery as pressure was mounted by the Postmaster General to hang men in chains for these crimes. The Admiralty Sessions also sentenced twenty-one men to hang in chains for the crimes of piracy, mutiny and stealing from a ship. The Admiralty Courts actually sentenced fewer men in total to dissection, and only murderers were sent to the anatomists; all those who received a post-execution punishment for crimes other than murder were gibbeted (TNA. HCA 1/61).

## HANGING IN CHAINS: SOME ARCHAEOLOGICAL AND HISTORICAL CONTEXT

While historians can start from the fact that the body of a criminal known from historical records must have been disposed of somehow, archaeologists, especially those working on earlier periods, start with the disposed body and work backwards to suggest that it might be the body of a criminal. In such cases the inference of criminality is mostly made when a body has been subject to non-normative mortuary treatment. The famous bog bodies of northern Europe are often exceptionally well preserved due to their deposition in the cold, anaerobic environment of the peat bogs. Many bog bodies date to the early modern period, and these are often interpreted as the victims of crime or misfortune (Turner 1995). The most celebrated bodies, however, are the Iron Age people whose deposition in the bog appears not to be accidental. Deviant burial in Britain during the medieval period has been the subject of extensive research recently, notably by Reynolds (2009), Buckberry (2008) and Cherryson (2008).

In the post-medieval period the bodies of criminals continued to be subject to special and unusual mortuary treatment. Criminals were often buried in special places, apart from the normative burial grounds of the parish. In Ireland this was usually in the ‘cillin’, a special burial place for the unbaptised and excommunicate, outside the control of the church (Donelly & Murphy 2008), and similar places almost certainly existed in parts of western Scotland and the south-west of England (Tarlow 2011, pp. 50–2). In most of England and Wales in the early modern period it might be burial in the less-favoured north side of the churchyard that signified social censure or, in special cases such as suicide, burial in the road was a widespread custom (Macdonald & Murphy 1990; Halliday 2010; although Houston 2011 contends that profane burial in the form of highway burial with a stake through the body was predominantly a south-east English custom, and that widely variable practices are described in provincial newspaper and legal accounts of the disposal of the suicide's body).

Unrelated developments in science converged with the history of punishment from the six-teenth century, when the growing demands of medical science began to be addressed by the legal provision of a small number of criminal bodies to the anatomists and surgeons of the universities. Archaeological evidence of early modern scientific anatomy survives at Oxford Castle where the remains of a number of individuals who had been subject to craniotomies or other cuts typical of the process of anatomical dissection were found buried in a disused moat (Norton 2006). The location of their burial place, outside any consecrated land; their orientation, along the line of the moat rather than west–east; the evidence of post-mortem cutting, and the lack of customary care in placing many of these bodies, some of which were buried prone, or had apparently been rolled into their grave from the side or had their limbs bound, all suggest that these were the burials of executed criminals. Evidence suggests that they were deposited between the fifteenth and the eighteenth centuries.

In the first half of the eighteenth century post-mortem provisions were part of the discretionary repertoire of the judge who might order that a felon be not only hanged but also dissected or ‘hung in chains’ — displayed in a gibbet. In 1752 a parliamentary act ‘For Better Preventing the Horrid Crime of Murder’ was passed which specified that no executed murderer should be allowed burial in consecrated ground, unless he had been first either dissected by the anatomists or ‘hung in chains’ (25 Geo II c.37: An Act for Better Preventing the Horrid Crime of Murder). This act answered to both a growing popular panic that murder, especially on the streets, was becoming more prevalent (King 2003), and to the much increased number of capital crimes (Linebaugh 1992; Gatrell 1994). By the end of the eighteenth century more than 220 offences carried the death penalty, including damaging the banks of canals and impersonating a Chelsea Pensioner. This compares to around fifty capital offences in 1688 (Potter 1993, p. 4). Death being pretty much the ultimate punishment possible for any offence, the only way to distinguish the punishment of the most serious among those crimes was to add what the Act described as ‘some further mark of infamy’ — and extend the punishment past the point of death.

Whether the criminal was to be dissected or hung in chains was not specified by the Murder Act and appears to have been left to the judge's discretion. When passing sentence the judge drew on tradition and expectations as well as guidance from the Act. A further layer of discretion came in after sentencing when local sheriffs might change the nature of post-mortem provision. We have been unable to determine what factors militated towards dissection rather than hanging in chains or *vice versa*. It is rare to find any discussion or reason behind the sentencing, though in one case

The learned judge, in passing sentence on murderers Richards and Smith in 1778, informed them that he should not conform to the ordinary practice in disposing of their bodies, but that after death they should be hung in chains, as a public spectacle of horror, til the fowls of the air and the vicissitude of the seasons had totally devoured and dissipated them (*Whitehall Evening Post*, 29 March 1788).

The only general trend is that capital crimes that threatened the State, such as piracy, smuggling, or robbery of the mail were more likely to be punished by the gibbet than ‘ordinary’ murders (King forthcoming). Particularly horrific murders such as multiple killings, murder after rape or killing children might also have been slightly more likely to result in a sentence of hanging in chains, although the classification of murders by degrees of horror is clearly neither an objective nor a pleasant task. It is certainly also true that many horrible murderers, pirates and very notorious criminals were dissected, and in some cases the criminal apparently considered this the worse post-mortem fate (Gatrell 1994, p. 192), although the general assumption appeared to be that hanging in chains was slightly worse (Dyndor forthcoming). Another significant factor seems to have been the demand from anatomists for bodies. No women were ever hung in chains, for example, and this is almost certainly because the executed bodies of young women, being much rarer commodities, were so highly sought-after by the surgeons that they could not be ‘wasted’ on the gibbet. Similarly, when two men were condemned for the same murder it was not uncommon for the older and stringier individual to be hung in chains while the younger and fitter one was sent to the dissection table; and the bodies of those of other races or with unusual physical conditions almost invariably went for dissection.

## THE PROCESS OF HANGING IN CHAINS

After the seventeenth century no criminal in Britain was gibbeted alive (Andrews 1899). Gibbeting was a post-execution punishment. Gibbeting alive was, however, practised in other parts of the world until the nineteenth century. In the eighteenth-century Caribbean, for example, rebellious slaves were sometimes gibbeted alive in an action somewhere between torture and execution, so that if the individual would agree to inform on others they might be released, but if they were too slow, or unwilling to do that, they would die in the gibbet cage (Burnard 2004, p. 151). This paper is mostly concerned with the gibbets of the long eighteenth century that were erected in Britain.

Most criminals were executed at a customary place of execution, often on the edge of a town, where all executions and often other public corporal punishments such as whipping took place. Between 1720 and 1830 198 people were hanged on specially erected scaffolds at the scene of their crime (Poole 2008). More than half of these crime scene hangings took place in the South-East (London and Surrey) and in Gloucestershire and Somerset. In his examination of the executions of the Gordon Rioters, Matthew White has suggested that this added to a sense of ‘local justice’ (M. White 2012, p. 208). Most of those executed at the scene of crime were taken down and disposed of elsewhere, but a minority were subsequently enclosed in gibbet cages and then hung up again on the same framework.

Execution took place either at the scene of the crime (Poole 2008; 2013) or more usually in the customary location of the scaffold. When a man was sentenced to be hung in chains the body would then be taken down and possibly prepared by painting with some kind of preservative. Secondary sources often mention the body being ‘dipped in tar’, but this is never mentioned in the sheriffs’ cravings, and there is no conclusive evidence that it happened, certainly not routinely. The body would then be fitted into its gibbet cage, often called ‘irons’ or ‘chains’, and transported to the place appointed for the gibbet. Unless the execution had taken place at the scene of crime, this was often many miles away — sometimes more than a day's journey, and required a cart and often a strong guard to prevent public disorder and to frustrate any attempt to rescue the body, all of which hefty expenses were detailed in the sheriffs’ cravings. Nearly all gibbets were structures made for a single individual and were not normally reused (except in the case of those sentenced by the Admiralty Court, whose normal practices were different to those described here and are discussed below). Once in the gibbet the criminal corpse could remain there for many decades, unless the body was stolen by friends or relatives — which was not infrequent despite the risk of transportation attendant on anyone caught removing a body from a gibbet —, or blown down, as happened to the gibbet of John Haines on Hounslow Heath (*Morning Chronicle*, Tuesday March 19, 1799). In most cases eventually the landowner or severe weather removed it. The Murder Act specified no minimum or maximum period of suspension.

## WHAT DID THE GIBBET LOOK LIKE?

The gibbet consisted of a tall wooden post — the sheriffs’ cravings often specify a height of 30 feet or more —with a cross beam at the top from which was suspended a short chain attached to an iron cage which contained the body of the criminal. These cages took various forms and range from the basic (a simple length of chain that ran between the criminals legs, together with a collar that bolted his head in position) to elaborate anthropomorphic cages with hinges, bolts and adjustable belts. The variety in form is explained by the infrequency of their manufacture which did not allow regional traditions to develop, and the great time pressure under which they were usually constructed (Tarlow 2014). The cage and chains were designed to allow the body to twist and sway in the wind, and to clank and creak as it moved. These noises, together with the flies and birds attracted by the decomposing flesh, would add to the sensory experience of the gibbet. Most gibbets were constructed for a single individual, but a very small number were made for two or even three. Aside from a few cases in London where bodies were hung on empty branches of a gibbet already in use, multiple gibbetings always involved the bodies of individuals convicted of the same crime and executed at the same time.

## GIBBETING ACROSS BRITAIN

Although the sentence of gibbeting was passed by the circuit judge who travelled between assize courts, hanging in chains was not practised equally frequently across Britain. London had far and away the most gibbetings. In part this is due to the fact that many of the most serious crimes were tried in London, even if committed elsewhere. London also had a huge population and well-known social and economic problems (Shoemaker 2004; J. White 2012). However, even in the rural provinces there were marked differences in the frequency of hanging in chains. In the counties of Sussex, Essex, Gloucestershire and Hampshire, for example, five or six gibbetings sometimes occurred within a decade, whereas in Cornwall there were none at all during the whole period and in County Durham there was only one. To some extent this mirrors the general disinclination of provincial courts to pass capital sentences and of the local administration to execute them (see King & Ward forthcoming). While there is considerable variety between even adjacent counties, in general it is true that gibbeting was more common in the South than the North, more common in the East than the West, and more common in England than in Scotland or Wales. At the time of writing our analysis of Scotland is incomplete, although Ph.D. student Rachel Bennet is studying post-mortem punishment in Scotland. We have undertaken no analysis of post-mortem punishment in Ireland although it is hoped that this will be the subject of future study.

## LOCATING THE GIBBET

The Murder Act does not specify any of the details of how or where hanging in chains ought to take place, but outside London it is almost universally traditional that gibbets are erected on the closest practical piece of land to the scene of the crime. There are some further requirements: the gibbet should normally be located in a place of high visibility and its erection should not cause ongoing inconvenience to travellers or local residents, though that last requirement was not always met, as will be shown below. These three requirements: proximity, visibility and practicality, will be considered in turn, and followed by a consideration of some exceptional categories such as the fate of those convicted for crimes at sea, or the murderers of London.

### PROXIMITY

The sentence passed on Thomas Smith and John Kennedy, convicted of highway robbery in 1787 in Hertfordshire, was unusual in recording the place of execution as well as of hanging in chains:

Let them be severally hanged by the neck until they be dead, at some open conspicuous place at a convenient distance from that where the said robbery was committed [Charleywood Common, Herts]. And I do hereby order that their Bodies shall be afterwards hanged in chains at the Place where the said execution shall be done (TNA. E389/248/67).

Newspapers often reported that bodies were gibbeted ‘at the place the fact was committed’. Both the provincial and London press provided details about the locations of gibbets. For example when Abraham Tull and William Hawkins were sentenced to hang, the *Whitehall Evening Post* announced that they ‘had the sentence of death passed on them by Sir Nash Grose, who ordered them to be executed on Mortimer Common, Berkshire (not far from the place where the murder was committed) on the Friday following, and their bodies to be afterwards hung in chains’ (*Whitehall Evening Post*, March 10, 1787 – March 13, 1787) (Pl. I). Another paper reported that the pair ‘were executed on Mortimer Common, near the place where they committed the murder, and their bodies afterwards hung in chains’ (*Bath Chronicle*, Thursday, April 5, 1787).

**Plate I.**
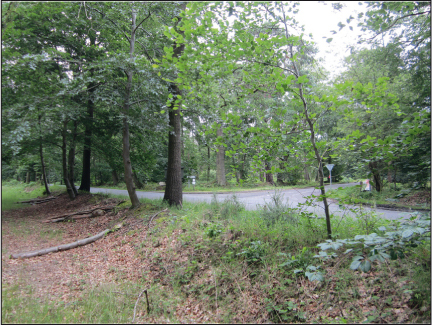
Mortimer Common, where Abraham Tull and William Hawkins were hung in chains in 1787

On some occasions the gibbet was located very close to where the criminal had lived. Where family members survived, however, the continuing presence of the decomposing body of their relative must have worsened the pain and prolonged the stigma. Brothers Richard and William Weldon were hung in chains in 1789 for the murder of the local butcher, near the lost village of Nether Hambleton, Rutland (now beneath Rutland Water), within sight of their family home. An estate notebook of 1797 annotated one cottage with a cross and the note ‘where you never go — mother to the young men that were hanged’ (Sleath & Ovens 2007, p. 205). The stigma of being related to a murderer was perhaps extended by enduring mnemonic presence of their actual gibbet close by. Benstead, convicted of murder in 1792, was hung in chains less than a quarter of a mile from his family's house near Lakenheath, Suffolk, but his body was taken down following requests from his family (*The Morning Post*, Tuesday, October 19, 1824).

Similarly, the family of Thomas Willdey, hung in chains on Witley Common near Coventry in 1734, petitioned the sheriff to have his gibbet taken down because the offensive nature of the body caused the family to suffer frequent ‘reproaches’ and the constant reminder to the community of the crime committed by Thomas Willdey made it difficult for his family to regain their former position in society or pursue their trades (TNA. SP 36/32/115).

Of the gibbetings that occurred after the Murder Act, the locations of at least seventy-six have been documented as being near to the place where the crime was committed. This was clearly the most important factor in determining where a body should be gibbeted, signalling that this form of post-mortem punishment, unlike dissection, was inextricably linked to the victim and crime. It appears that across the whole of Britain gibbet locations were often chosen for their proximity to the scene of crime. Where it was impractical for the gibbet to be put there, it would be positioned as near as possible to the desired location. We have been unable to be any more precise about the actual distances involved because our sources for both the location of the crime and the location of the gibbet are generally imprecise: ‘on the common’ and ‘close to the scene of crime’ being typical locators.

### VISIBILITY

Another important factor that contributed to the positioning of the gibbet was how visible the body would be to local people, travellers and the crowds who assembled when a gibbeting took place. Gibbets were often very high structures: the cravings regularly specify a height of 30 feet or more. The gibbeted body was thus not only safe from interference, but highly visible to people passing by, even at a distance of several hundred metres. Use was sometimes made of natural eminences or elevations in order to maximise the conspicuity of the gibbet from nearby roads. Anthony Lingard was gibbeted at St Peter's rock in the Derbyshire peaks (Pl. II). Not only is this clearly visible from the toll-keeper's cottage where he committed murder, and therefore also close to the toll road, it is marked by a large natural rocky outcrop which was a well-known feature in the local geography. When William Lewin was hung in chains in Cheshire in 1788 the place selected was the top of Helsby Tor, about 8 miles from Chester, from where, according to the author of a pamphlet about his crime and trial, ‘it may be conspicuously seen, and, by means of glasses, is visible to the whole county, most parts of Lancashire, Shropshire, Derbyshire, etc., etc.’ (*Trial of William Lewin*, Chester 1791). Even allowing for some exaggeration, the selection of a very visible situation for the gibbet was clearly important here.

**Plate II.**
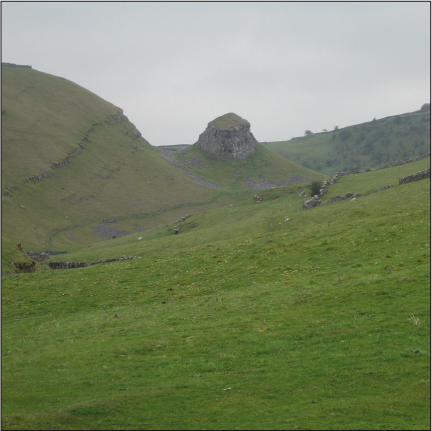
St Peter's rock, Derbyshire, where Anthony Lingard was hung in chains

Archaeologists and landscape historians have noted the widespread medieval practice of executing criminals on or adjacent to archaeological features, especially prehistoric burial mounds *(e.g*. Whyte 2003; Reynolds 2009; Meurkens 2010). In the post-medieval period such practices are not widely known in the British Isles, although there are a couple of examples. The murderer Loseby was hung in chains on top of a tumulus on the Watling Street Road, about 4 miles from Rugby. The tumulus was subsequently destroyed in the construction of the Daventry to Lutterworth turnpike in the late eighteenth century (Bloxam 1875). Human bones discovered in the gardens of adjacent houses were thought at the time of their excavation in the nineteenth century to represent the remains of people hanged and buried at the crossroads, and it may be that in Loseby's case the crossroads location was more significant than the antiquarian nature of the site. The tumulus might well have been selected only as a convenient elevated spot.

The positioning of the gibbet by the roadside was significant and some historians have suggested that gibbets were placed at crossroads or boundaries (Whyte 2003) or along highways (Gatrell 1994, p. 267). Placing the gibbet by the road obviously allowed for viewing by greater numbers of people. In 1823 John Rolfe's body was hung in chains on a ‘lofty gibbet near Littleport turnpike Road’ (*The Morning Chronicle*, Friday, February 28, 1823). Rolfe had been convicted of murdering John Landen with a hedge-stake, but men convicted of highway robbery or mail robbery were equally likely to be hung by the roadside. Spence Broughton was gibbeted at the side of the road where he committed highway robbery in 1792. Likewise John Price was hung at the junction of a major coach route in Oxfordshire. Situated by the Three Pigeons public house, his gibbet was designed to act as a warning to other highway robbers who used the road (Pl. III). These locations fulfilled both desirable criteria of being close to the scene of crime and very visible.

**Plate III.**
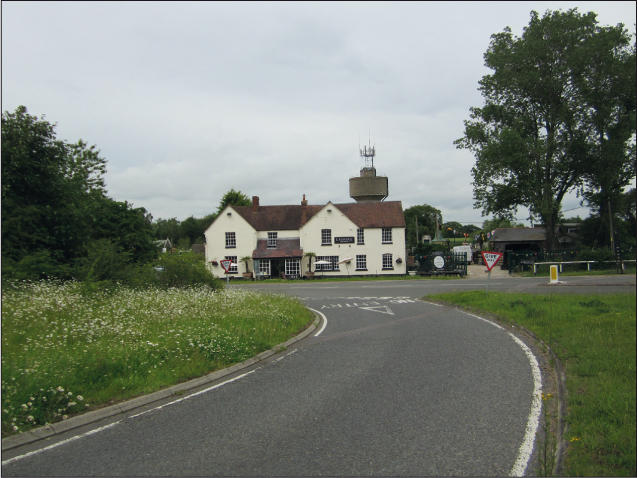
John Price's gibbet was situated by the old Three Pigeons pub, close to the road junction

If some gibbets were to be visible from the road, there were others that were to be visible from the sea. Gibbets along the coast or at ports were to deter maritime crime. The ‘Thames tassels’, Admiralty gibbets along the Thames estuary, were visible to all visitors arriving in London by sea, and one author noted that in 1841 there were many people who could still remember the sight of the gibbets opposite Blackwall waving in the wind, ‘“a gibbet's tassel” one of the first sights that wont to greet the stranger approaching London from the sea’ (Knight 1841, p. 360). Away from London, the port at Portsmouth was extensively used as a place for gibbeting. Between 1760 and 1784, six bodies were hung in chains in the area, including ones at Gosport, Cumberland Port and Blockhouse Point. The most infamous of these was the arsonist James Hill, otherwise known as Jack the Painter, who attempted to burn down the port as a protest for American Independence. His gibbet was later used as a reference point, such as when midshipman Murphy was executed and the paper reported that ‘After hanging the usual time, his body was cut down and buried under the gibbet where John the Painter hangs in chains’ (*General Evening Post*, June 22, 1779). In Devon, Smith and Richards were gibbeted by the Church on the marshes. Similarly Michael Curry's body was hung in chains on the coast overlooking St Mary's Isle, Northumberland.

### PRACTICALITY

Executions and post-execution punishments were throughout this period the focus of great public interest. While the numbers who could see a dissection underway were ultimately limited by the capacity of the building, and controlled by policing access, the crowd at an open gibbet could, and frequently did, run into tens of thousands. An estimated 40,000 people attended the first day of Spence Broughton's gibbetting near Sheffield (*The True and Ilustrated Chronicles* 1900, p. 6), and a similar number was cited for William Smith's gibbeting on Finchley Common in 1782 (*Public Advertiser*, April 30, 1782) (below). Therefore, gibbet sites were favoured which allowed the crowd to gather without altogether halting traffic, trampling crops, distressing animals or damaging property. Wide road verges, waste ground or, best of all, open common were therefore the best locations from a practical point of view. The sheriff who was planning the gibbeting was also ultimately responsible for public order and often had to spend money on guards to patrol the crowd and to accompany the body to prevent its rescue. There is, however, no evidence that the sheriff 's financial concerns affected the choice of site.

For certain crimes it was not practical to meet all the criteria — especially proximity to the scene of crime. Where the crime had occurred in a densely populated area such as London, or at sea, other strategies were developed, which will now be briefly considered.

In the case of criminals sentenced to hang in chains for crimes committed at sea there were obvious practical reasons why the gibbet could not be located at the scene of crime. Such crimes were normally tried by the Admiralty Courts in London and criminals sentenced to death there were customarily executed at Execution Dock. From there the bodies of those sentenced to hang in chains might be removed to an appropriate location around the coast, or along the Thames estuary. The majority of those sentenced to hang in chains by the Admiralty Courts had committed crimes on the high seas which had no local connection to any part of the British coast. Their bodies were gibbeted along the Thames estuary where they were visible to the huge ship traffic in and out of London. This was presumably intended to act as a warning to would-be pirates, mutineers and murderers, but they were hard to avoid for other travellers too. A letter from Mr Dykes, ‘a private individual’, to Robert Peel as Home Secretary in 1824 complained about the continuing presence of gibbets and their contents along the banks of the Thames, upsetting ladies, foreign travellers and others. He expresses the view that such a sight is ‘revolting, disgusting, pitiable, dishonourable to the law's omnipotence, and discreditable to the administrators of the law’ (TNA. HO 44/14/87). Dykes's concerns can be understood when one sees the number of gibbetings of pirates. Between 1814 and 1816 alone, six men were hung in chains along the estuary for murder on the high seas, according to the Admiralty Sessions. This was at a time when the practice had largely fallen out of use in all other contexts. There were in fact only two other gibbetings in the whole decade 1810–1820 in the whole of England and Wales.

In general, in London the location of gibbets followed a different pattern to the rest of the country. Rather than being placed near to the location of the crime, gibbets were erected in specific areas. There were a number of gibbet areas just outside of the capital. These were places associated with crime, execution or hanging in chains. Finchley Common, Hounslow Heath, Shepherds Bush, the Edgware Road and Stamford Hill were all used on multiple occasions. At one stage the Edgware road was described as being ‘littered’ with gibbets (*Lloyd's Evening Post*, April 4, 1763). In 1764 it was reported that ‘Saturday night last the only remaining gibbet at Shepherds Bush was blown down, so that place remains now without any marks of ignominy upon it; which has not before happened for a century past’ (*Gazetteer and New Daily Advertiser*, Tuesday, March 26, 1765). For this reason London and Surrey were the only places in which gibbet posts were reused. On Stamford Hill the smuggler Arthur Gray was hung on the post where Hosea Youll had been gibbeted. William Corbett in 1764 was placed on a ‘vacant gibbet’ and Gill Smith was hung on a spare limb of the gibbet from which two of his colleagues in crime had been hung in 1738. There are bodies that are placed near where crimes were committed in London, though these tend to be in the places that gibbets were commonly placed.

### SENSIBILITY

Meeting the need for the gibbet to be sited conspicuously and close to the scene of crime would cement its association with the serious crime, maximise its symbolic value as a deterrent, and enhance the State's display of authority. But the horror of the spectacle, and the sensory impact of decaying bodies, were also often incompatible with modern sensibilities. Thomas Watkins's gibbet was originally placed at the corner of Gallows Lane in Windsor; however two months later the body was removed and placed by the river due to complaints that it was a ‘nuisance to passengers’ (*St. James's Chronicle or the British Evening Post*, May 24, 1764). Similarly the body of John Swan was removed from one part of Epping Forrest to another as

a new order came from Mr Justice Wright, directing that the body of Swan should not be hung in chains there it being in full view of some Gentlemen's houses on the Forest, but left to the Gentlemen of Walthamstow to consult with the under-sheriff, and fix a proper place to erect a gibbet on; whereupon it was agreed, that Buckets Hill, near the Bald Faced Stag, was a proper place, not only in situation, but it being a place where Mr Jeffreys [the victim] often resorted from whence Swan used to fetch him (*General Advertiser*, Tuesday, May 5, 1752).

The new location was both remote from the offended sensibilities of the ‘Gentlemen’ and symbolically resonant because it was still a meaningful place in the story of the crime.

There were thus numerous factors that led to the decision to place a gibbet at any chosen location. It is significant that there was a balance between utilising locations that related to both the crime/criminal and the landscape. Furthermore, the suitability of the location needed to be considered, especially if there was a risk that it would cause distress to local people or travellers. It is worth noting that gibbet locations were not necessarily permanent, and could be moved if the location was not right.

## THE GIBBET IN THE LANDSCAPE

The gibbet was a large, unsightly structure that had an impact on the area in which it was placed with the spectacle, smells and atmosphere it created. Both in the short and long term, the gibbet affected the landscape in which it was situated.

### GIN AND GINGERBREAD: THE CARNIVAL OF THE GIBBET

The place of the gibbet was a strange intersection of moral education, demonstration of State power, and public entertainment. The new gibbet attracted huge crowds — estimates of 40,000 people a day are not unusual in contemporary newspaper reports. Such large numbers attracted entrepreneurial individuals who came to sell food and drink. The gibbet became a place for socialising and games: in the newspapers, shocked commentators wrote of the unseemly resemblance to a fair. When the two murderers Conoway and Richardson were hung in chains on Bow Common in London in 1770, the newspapers were outraged by the behaviour of the gibbet crowd:

The place where Conoway and Richardson hang in chains still continues a perfect fair. A booth is erected, and several kinds of diversions carried on, even under the gallows. Several people have climbed up the gibbet, and some of them taken the caps from the malefactors’ faces. One fellow had the hardness to call out “Conoway, you and I have often smoked a pipe together, and so shall we again” on which (to no small diversion to the mob) he climbed up the gibbet with two lighted pipes, one of which he stuck in Conoway's mouth, and the other he smoked as he sat across the gallows. It is a pity the magistracy did not exert themselves on this occasion (*General Evening Post*, 2 August, 1770)

When the gibbets had first appeared there two weeks earlier, the *Public Advertiser*, another London paper, complained that ‘drinking booths were erected, and the place had the same appearance of general disorder as a fair day’ (*Public Advertiser*, 24 July, 1770). The same newspaper was still indignant twelve years later, when another London murderer, William Smith, was gibbeted on Finchley Common in 1782. Smith's body was the central attraction for more than 40,000 people who came in ‘coaches, chariots, phaeton &c’ as well as on foot to enjoy fried sausages or, for those of less opulent means, gin and gingerbread in a scene of ‘shameful riot and disorder’ (*Public Advertiser*, 30 April, 1782).

Outside London, the attraction of a gibbeted criminal was just as great. Since gibbeting happened relatively infrequently in the provinces, and gibbeted criminals were normally notorious villains whose crimes and trial had been extensively reported and commented on locally, there was great public interest in their execution and display. When Tom Otter was gibbeted in Lincolnshire in 1806, there was ‘a week of merry-making of the most unseemly character’ when large crowds were catered for and booths erected, presumably for food and drink (Andrews 1899, p. 68). Huge crowds and a carnival atmosphere, together with the infrequency of gibbeting outside London, would combine to sear the event of hanging in chains into local memories. Passing the place of a gibbet would be an occasion for recollecting the execution and gibbeting, and retelling the story of the crime and the criminal. The abjection of the body gave force and endurance to the fame and notoriety of the individual.

Why did a gibbet attract such a carnival crowd? While the establishment hoped that maximum numbers would be exposed to the powerful threat of the gibbet, and would respond to its presence as a warning and a moral lesson, the crowd also responded to the transgressive thrill of the corpse. The carnivalesque appeal of the execution itself, and of anatomical dissection (especially in early modern Europe) have been noted elsewhere (for the former, see Laqueur 1989; Gatrell 1994; for the latter Ferarri 1987). The excitement about the gibbet fed upon and fed into the growing cult of criminal celebrity. As Penfold-Mounce (2010) has recently explored, there is a notable association between social transgression as manifest in the body of the criminal, and fame. The criminal body and its parts became sought-after relics or curios, touched by the ‘glamour’ of the criminal (Tarlow forthcoming).

### LANDSCAPE WITH GIBBET

The carnivals and crowds did not last as long as the gibbet did. As the months and years passed, the body decayed and parts of it fell and were taken by animals (or sometimes, by relatives of the deceased — Eugene Aram's wife is reputed to have secretly collected parts of her husband's body as they fell, and buried them: Scatcherd 1838). However, gibbets were prominently and conspicuously sited and had other functions in the landscape. They became known as landmarks or even took on functions as boundary markers. When the 1628 gibbet of John Felton, murderer of the Duke of Buckingham, fell down it was replaced by an obelisk in 1782 — not as a memorial to Felton or the Duke of Buckingham, but to serve as a boundary marker as the old Southsea gibbet had come to mark the boundary of the borough of Portsmouth. The obelisk has since disappeared (Memorials in Portsmouth. co.uk).

Some places took their names from the presence of a gibbet: Combe Gibbet (Berkshire), Caxton Gibbet (Cambridgeshire), Winter's Gibbet (Northumberland), for example (Dyndor forthcoming); and many criminals gave their names to the roads or fields on which their gibbets stood: Lincolnshire's Tom Otter's Lane (adjacent to which are Gibbet Wood and the Gibbet Lane cottages) and Ralph's Lane both remember gibbeted criminals in their toponyms, as does Old Parr Road in Banbury. The road in Attercliffe on which Spence Broughton's gibbet stood is now called Broughton Lane. There are dozens of Gibbet Lanes, gibbet hills, gibbet farms and gibbet woods in England. Not all of them date to this period: indeed, probably most are late medieval or early modern in origin, but some are certainly traceable to particular gibbeted individuals, such as the Gibbet Hill Lane (Pl. IV) just north of Scrooby, Nottinghamshire, which marks the location where John Spencer was gibbeted in 1779 near the toll bar where he murdered the keepers of the toll gate. Some places named after gibbets have retained the name, but knowledge of the origins of that name have faded from memory. Gibbet Hill is a well-known place at Warwick University, though it would be difficult to find someone who remembered Edward Drury, Robert Lesley and Moses Baker who were hung in chains there in 1764 (Warwickshire Directory 1874). The gibbet has been immortalised in the landscape in the name of Gibbet Hill. Similarly Curry's Point, Whitley Bay, is named after the criminal hung in chains there, according to its blue plaque detailing the history of the site.

**Plate IV.**
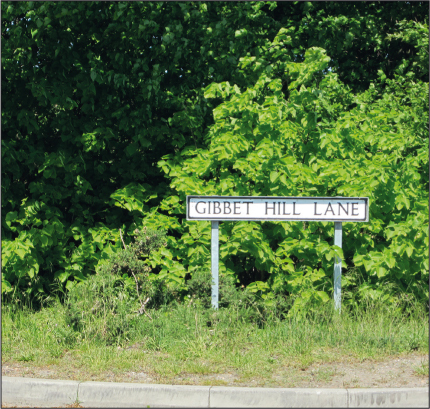
Gibbet Hill Lane, Scrooby, Nottinghamshire

The presence of a gibbet affected the experience of passing through a locality. Their utility as landmarks did not necessarily outweigh the unpleasantness of proximity to the smells and sights of bodily decay. John Spencer's body at Scrooby produced such a stench when a member of the local militia shot it (the implausible story was that he had mistaken the body for a deserter) that people were unable to pass through the area for several weeks (Sutton 1850, pp. 35–6).

In the absence of any legally specified term for which the body must remain on the gibbet, bodies were generally left until weather, land development or circumstance brought them down. Accordingly, a gibbet might remain standing for many years or even decades. Spence Broughton's gibbet-post with irons, skull and some bones and rags, was still standing nineteen years after it had been erected. According to Andrews it was removed in 1827 or 1828 when the landowner became fed up with sightseers trampling his land and wished to enclose the part of the common with the gibbet on (Andrews 1899, p. 16).

Recurrent descriptions of birds’ nests in the bones hanging on the gibbet suggest the longevity of the structures. One man found a starlings’ nest in the ribs of Gabriel Tomkins twelve years after he had been gibbeted and ‘was obliged to break one of the ribs to get them out’ (*St. James's* *Chronicle or the British Evening Post*, 22 June, 1762). There are also several reports of gibbets that have been affected by the weather which indicate how long the gibbet was standing. For example a letter from Derby shows that Matthew Cocklane's gibbet was *in situ* from 1776 to 1791:

One day last week, a lad was met coming into this town, having in his hand the skull of Matthew Cocklane, who was executed on the 21st March, 1776, for the murder of Mrs Vickars, and afterwards hung in chains. It seems that the wind had blown him from his exalted situation the preceding night. His hair, skin and most of his bones were in high preservation. Numbers, who had often stood in melancholy gaze, repaired to the gibbet, and returned with various parts of his remains (*Lloyd's Evening Post*, October, 1791).

The posts themselves could last far longer than the remains of the body, as shown by the story of the gibbet post erected in 1773 for the body of Edward Corbet:

The last remains of the Gibbet, which had been used as a gatepost for the rickyard belonging to Mr Dockens of Bierton, was taken down on August 15th, 1860. The post was about six feet long, and had been cut from the upper part of the Gibbet, and was about six inches square; in it were two mortise holes, the one eighteen inches below the other; there appear marks of the rubbing of chains. Mr Watts, chairmaker, of Bierton, bought the piece with a view to work it up into various fancy articles (Lambourne: Centre for Buckinghamshire Studies: D 15/9/4).

## THE LEGACY OF THE GIBBET

Gibbet posts were a transient part of the landscape. Perhaps several of them have left no enduring mark of their presence either visible or in the mind, but many have been remembered through lasting memorials to the victims, or signposts marking the gibbet. One of the most famous memorials at a gibbet location is that at Hindhead Common, Surrey. ‘The Sailors Stone’ marks the spot where, according to the inscription on the stone, three men were hung in chains for murdering an unknown sailor. In a more makeshift fashion, Jack Upperton's gibbet in Wepham Wood, Surrey has been marked by a post with the date of his execution on it (Beaumont 2006). The Noose and Gibbet pub in Attercliffe is a modern creation but has fabricated a relationship to the nearby former location of the gibbet of Spence Broughton. The modern Noose and Gibbet took inspiration from the history of the locality, and the name of the Broughton Road on which it stands, and is decorated with fictive ‘Wanted’ posters, and a cage with a figure of a man hangs outside (a living man in a kind of animal cage rather than a replica of Broughton's gibbet) (Pl. V). Apparently gibbets can still mean gin and gingerbread.

**Plate V.**
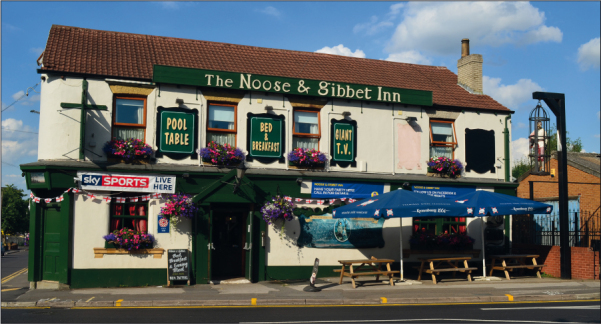
The Noose and Gibbet pub, Broughton Road, Sheffield

In Congerstone, west Leicestershire, a weathered wooden post by the side of a rural road is in fact the last surviving *in situ* original gibbet post (Pl. VI). It is marked by an interpretation board featuring an improbable artistic reconstruction of a hanging in chains. But in other places the local inhabitants had become so attached to their gibbets that the gibbet posts have been replaced — sometimes many times — to give continuity to the location. Coombe Gibbet and Caxton Gibbet are both sites which still have replicas of the former posts, though there is again limited contemporary knowledge of the individuals gibbeted there. It is supposed that the original gibbet at Coombe dates back to 1676, when Dorothy Newman and George Bromham were hanged for murder, however it is unclear whether either were hung in chains there. The gibbet currently standing is in fact the seventh on that site (Hungerford Virtual Museum.co.uk). Interestingly the twentieth-century history of Combe Gibbet evidences both great local attachment in the repeated re-erections of a gibbet at that site, and the potency of the structure as a site for political protest: two of the gibbets were deliberately cut down in protest against capital punishment. On 18th June 1970 the *Newbury Weekly News* records that it remained a location of political action: ‘Combe Gibbet, scene of a stunt last week when an effigy bearing political posters was hung from it, came in for more election fun this week when the Labour candidate, Mr Tim Sims, placed flowers there ‘in memory of the past injustice of a feudal system, brought to an end by a compassionate society and the never-ending fight by the common man for equality’. A final replica gibbet post at Steng Cross, Northumbria, is called Winter's gibbet after the man hung there in 1792. Both the story of William Winter and the gibbet post remain part of the local history of Rothbury (Rothbury.co.uk) and that gibbet has also been re-erected several times.

**Plate VI.**
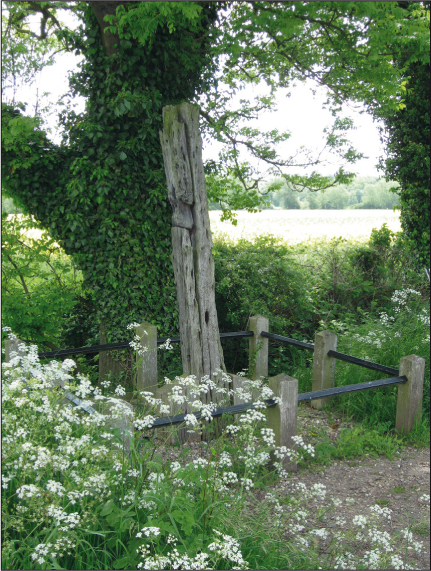
Congerstone gibbet post, Leicestershire

The places of the gibbeted dead were populated not only with the curious living but also sometimes by the ghosts of the dead. One ghost story notes that ‘a skeletal apparition of a man still trapped in the gibbet, which displayed his corpse, was said to haunt Gibbet Hill, Beacon Edge, Penrith’ (www.mysteriousbritainandireland.co.uk ). On Durdham Down in Bristol the ghost of William Jenkin Prothero, gibbeted there in 1783, allegedly descended from his hanging corpse each night at midnight to haunt Clifton. It is supposed that his sentence was modified so that his body could be removed from the gibbet and buried, as his ghost caused such alarm (www.bristolinformation.co.uk 2014). The attachment of ghost stories to gibbeted men is unsurprising: hanging on a gibbet, creaking and swaying in the wind, the bodies hung in chains would have created an eerie atmosphere that lent itself to tales of the supernatural. Owen Davies and Francesca Matteoni, whose research into the folklore of the criminal corpse is another thread of our project, have noted that remarkably little ghost lore attaches to urban places of execution or gibbeting; nearly all the ghosts at gibbets are in rural and provincial locations (pers. comm. Owen Davies). Davies (2007) has suggested that there were in general fewer accounts of urban ghosts than of their rural counterparts, and that urban hauntings were generally linked to houses and churchyards.

## CONCLUSIONS

Gibbet places persist in the toponyms of England and in folk histories. Hanging in chains was intended as a punishment worse than death — a ‘further mark of infamy’, but it was also an alternative to the other great post-mortem punishment of the eighteenth century: anatomical dissection. Although the Murder Act presents these two punishments as equal options, they actually have quite different tendencies. Anatomical dissection ultimately anonymises and annihilates the body. The particular criminal — and the particular crime — are irrelevant to the fate of the body, which comes to stand for a universal medical body. After it had been cut down to the bones, the parts divided and examined, there was rarely even enough body to bury. The anatomised criminal body was fully dislocated from any particular place and ultimately obliterated. By contrast, the criminal hung in chains was written enduringly into the landscape. His name often became inalienable from the place of his gibbet, and his particular crime and fate was remembered through the erection of what was, effectively, a monument or even a Benthamite ‘auto-icon’. The gibbet functioned as a mnemonic; the massive crowds and carnival at the occasion of its erection served to make the occasion unforgettable in local memory.

Moreover, the two punishments also have different genealogies: gibbeting coming out of the late medieval tradition of bodily punishments which reveal the triumphalist State and the humiliated deviant. It goes with heads on spikes and nailed-up quarters. Dissection, though equally dreaded, comes from the growth of scientific medical knowledge, and did not originate primarily as a punishment at all. In making anatomical dissection a part of the punishment of criminals, however, the suspicion that the anatomist was in collaboration with the hangman grew (Forbes 1981; Richardson 1989).

Given these divergent histories and consequences, it is remarkable that such different practices were ever considered alternatives of equal weight. This demonstrates not only the distance between the public perception of the nature of anatomy and the emic perspective of its practitioners (as discussed by Richardson 1989), but also perhaps a failure on the part of the legislature to consider the immortality that sometimes accompanies notoriety.

Finally the study of the gibbet illustrates one of the paradoxes of the cultural history of the body: that the dead body can be both the epitome of powerlessness, upon which any design of the living can be enacted, and simultaneously very powerful, maintaining a presence in the places, fears and imaginations of the living for many generations after. There is no evidence that the erectors of gibbets gave much thought to the possibility that a gibbet's presence would permanently affect the landscape. Their siting was intended to maximise the impact of retributive and deterrent justice, enacted on the body. Yet these historically situated moments of punishment had unanticipated long-term consequences, attaching the communal memory of atrocity and social vengeance to the enduring features of the landscape. A declaration of State power accidentally enhanced the glamour of the celebrity criminal, given mnemonic force by the gibbet in the landscape.

## Figures and Tables

**Table:** PRIMARY SOURCES

ECCO	Eighteenth Century Collections online (http://gale.cengage.co.uk/produced-highlights/history/eighteenth-century-collections-online.aspx).
Lambourne	Centre for Buckingham Studies D 15/9/4
TNA	The National Archives, Kew

## NOTES

1. All data derived from the work of the Criminal Corpse project will be available on our website following completion of the research, in the form of a database. Any enquiries should be made to the authors.
